# 

**Published:** 1909-01-15

**Authors:** 


### Dental Register

LIBRARY
GENERAL ...
FEB 1 1909

NELVILLE S. HOFF, Editor,

Ann Arbor, Mich.

Vol. LXIII — JANUARY, 1909 — No. 1

SAMUEL A. CROCKER & CO., Publishers
OHIO DENTAL AND SURGICAL DEPOT
35 W. FIFTH STREET, CINCINNATI, O.

PRICE, ONE DOLLAR A YEAR

Entered at the Postoffice at Cincinnati, O as second-class mail matter

